# Dissecting the hemagglutinin head and stalk-specific IgG antibody response in healthcare workers following pandemic H1N1 vaccination

**DOI:** 10.1038/npjvaccines.2016.1

**Published:** 2016-07-28

**Authors:** Sarah M Tete, Florian Krammer, Sarah Lartey, Geir Bredholt, John Wood, Steinar Skrede, Rebecca J Cox

**Affiliations:** 1 Department of Clinical Science, The Influenza Centre, University of Bergen, Bergen, Norway; 2 Department of Clinical Science, KG Jebsen Centre for Influenza Vaccines, University of Bergen, Bergen, Norway; 3 Department of Research & Development, Haukeland University Hospital, Bergen, Norway; 4 Department of Microbiology, Icahn School of Medicine at Mount Sinai, New York, NY, USA; 5 National Institute for Biological Standards and Control, Hertfordshire, UK; 6 Section for Infectious Diseases, Haukeland University Hospital, Bergen, Norway

## Abstract

Traditionally, neutralising antibodies that are directed to the major surface glycoprotein hemagglutinin (HA) head domain are measured as surrogate correlates of protection against influenza. In addition to neutralization, hemagglutinin-specific antibodies may provide protection by mediating antibody-dependent cellular cytotoxicity (ADCC). During the 2009 pandemic, vaccination induced HA-specific antibodies that were mostly directed to the conserved HA stalk domain. However, the protective role of these antibodies has not been investigated in detail. We quantified the HA head and stalk-specific antibodies, their avidity, ability to neutralise virus and activate natural killer cells in an ADCC assay. We analyzed sera obtained from 14 healthcare workers who had low hemagglutination inhibition (HI) antibody titres at 3 months after pandemic H1N1 vaccination as well as from 22 controls. Vaccination resulted in a HA stalk dominant antibody response in both low responders and controls. Revaccination of low responders, 5 months later, resulted in a boost in antibodies, with HA head-specific antibodies dominating the response. Comparative analysis of head and stalk antibody avidities revealed that stalk-specific antibodies were qualitatively superior. Furthermore, stalk-specific antibodies mediated virus neutralization and had significantly higher ADCC activity than head-specific antibodies. Despite the head and stalk-specific antibodies being lower in low responders, they had comparable antibody avidity, ADCC functionality and neutralising capacity to those of controls who had high HI titres post-vaccination. Thus, our study has demonstrated that HA stalk-specific antibodies may have an important role in protection through neutralization and ADCC in low responders who do not maintain seroprotective HI antibodies.

## Introduction

Influenza pandemics occur at unpredictable intervals when a novel influenza virus arises which can place a major strain on the global healthcare system. These pandemic viruses can cause high levels of severe illness and death. In 2009, an influenza A H1N1 virus strain caused a pandemic that started in Mexico and California then rapidly spread globally. The pandemic H1N1 (H1N1pdm) strain was antigenically distinct from the recently circulating seasonal H1N1 strains and the majority of the population was immunologically naïve to this virus.

Annual influenza vaccination is recommended for healthcare workers (HCW) so as to maintain the integrity of the healthcare system, reduce absenteeism and reduce influenza A transmission to vulnerable patients.^1^ Vaccination of HCWs has been shown to protect hospitalised patients as well as decrease influenza-like illness and mortality in residents of care-facilities.^2^ During the 2009 pandemic outbreak, the World Health Organization prioritised HCW for vaccination. H1N1pdm vaccination studies showed that a single dose of pandemic vaccine elicited protective serum hemagglutination inhibition (HI) titres in adults, including HCW.^3–8^ However, seasonal influenza vaccines did not induce protection against the novel H1N1pdm virus.^9,10^

HI antibodies are directed to the major surface glycoprotein, hemagglutinin (HA), and are the primary correlate of protection. HA is synthesised as a precursor, HA0, which is then cleaved by host proteases into disulphide-linked HA1 and HA2 subunits, activating virus infectivity.^11^ Antibodies directed to the HA head domain that is composed of the majority of the HA1 subunit prevent virus attachment to the sialic acids on host cells. These antibodies directed to the immunodominant head of the HA have potent neutralising activity that can be detected by HI or microneutralization assays. Antibodies directed to the HA stalk domain, primarily composed of HA2 subunit and the N- and C-terminal ends of HA1, have other functions, including blocking viral fusion with the host cell and antibody-dependent cellular cytotoxicity (ADCC).^12^

H1N1pdm vaccines preferentially induced HA stalk-specific antibodies. In contrast, seasonal inactivated vaccines induce strain specific antibodies directed to the HA head domain and minimal HA stalk-specific antibodies.^13,14^ Furthermore, HA stalk antibodies are postulated to be boosted most efficiently in individuals previously exposed to HAs whose head domains differ substantially from the infecting novel virus strain. Here, a memory B-cell response is boosted against the conserved HA stalk domain.^15^ Importantly, HA stalk-specific antibodies are broadly reactive and may have a significant role in protection against infection in the absence of HA head-specific antibodies.

In this study, we analyzed the magnitude of HA-specific antibodies induced after adjuvanted pandemic influenza vaccination in HCW. We also analyzed the quality as well as the neutralising and ADCC function of HA-specific antibodies in low-responder HCW who fail to maintain seroprotective HI responses after H1N1pdm vaccination.

## Results

### Low responders failed to maintain HI titres post-vaccination

**Figure 1:**
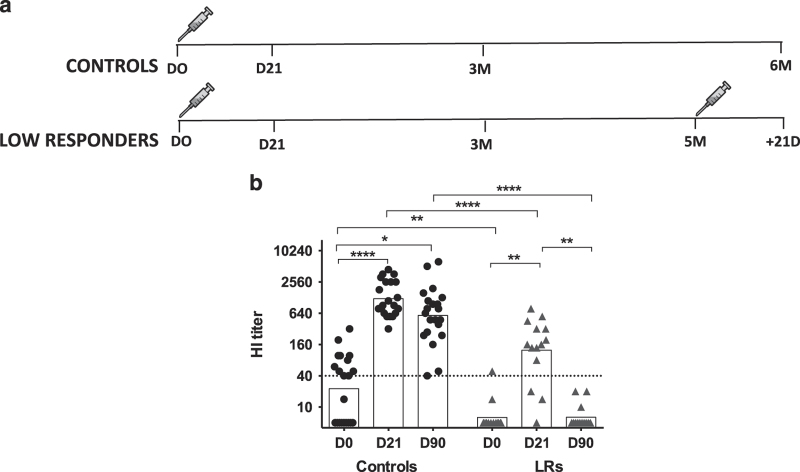
Study outline. (**a**) Thirty-six HCW received the AS03 adjuvanted monovalent pandemic influenza vaccine in 2009. Serum samples were collected before vaccination (D0); D21, 21 days, 3M and 6M post-vaccination. On the basis of HI titres at 3M, 14 HCW who did not have seroprotective titres (low responders) were offered revaccination. 12 HCW were revaccinated 5M after the first vaccination. Additional serum samples were collected at time of revaccination (5M) and 21 days later (+D21) in low responders. (**b**) HI titres against influenza A H1N1pdm virus at D0, D21 and 3M. The dotted line indicates a titre of 40, which is considered protective. * indicates statistically significant differences in responses *P*<0.05; **P<0.01; ***P<0.001.

Thirty-six HCW were recruited to the study based on their HI response and split into two groups; low responders (LRs) who failed to maintain protective HI titres by 3 months (3M) and a control group (Figure 1a). Fifty per cent of the controls had protective HI titres (geometric mean titre (GMT)=23) before vaccination in comparison to 1 LR (7%) (GMT=6). Following pandemic H1N1 vaccination, HI titres increased significantly by D21 in both groups (*P*<0.01). However, in LRs, titres were significantly lower (GMT=132) (*P*<0.001) than controls (GMT=1,223). All the controls maintained their protective HI titres at 3 mol/l, whereas HI titres decreased below protective levels for all the LRs (Figure 1b). This decrease may imply that LRs are no longer protected, although their antibodies may mediate protection through other mechanisms.

### The back-boost of cross-reactive antibodies to pre-pandemic seasonal H1N1 strains is lower in LRs

**Figure 2:**
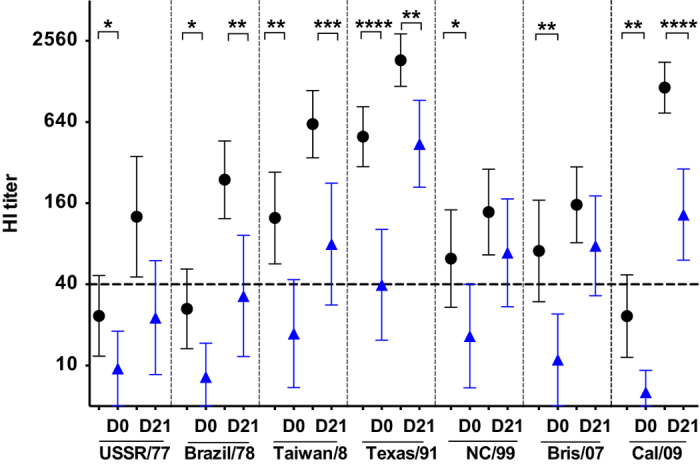
HI titres to previous seasonal H1N1 viruses. The HI assay was used to examine the cross-reactive HI responses at D0 and D21 against six historical H1N1 virus strains; A/USSR/90/77 (USSR), A/Brazil/11/1978 (Brazil), A/Taiwan/1/86 (Taiwan), A/Texas/36/91 (Texas), A/New Caledonia/20/99 (NC) and A/Brisbane/59/07 (Bris). The HI response to the H1N1pdm(Cal) virus is shown in the last column. The black circles represent the GMTs for controls, whereas the blue triangles represent the GMTs for LRs. The error bars on the plot show the lower and upper extremes. *P<0.05; **P<0.01; ***P<0.001, ****P<0.0001.

The H1N1 strains circulated from 1977 to 2008, when the H1N1pdm appeared and replaced them as the seasonal strain. We examined the cross-reactivity of the HI response in 20 controls and 14 LRs to the following historical strains; USSR/77, Brazil/78, Taiwan/86, Texas/91, New Caledonia/99 and Brisbane/07 (Figure 2).

Interestingly, pre-vaccination, LRs had significantly lower titres (GMTs<40) to all the seasonal H1N1 strains tested in this study (*P*<0.05) than the equivalent responses to A/California/07/09. This was in contrast to controls who had HI GMT>40 for all strains except for the oldest strains (USSR/77 and Brazil/78). Of the controls, 70–100% had seropositive pre-vaccination titres to Taiwan/86, Texas/91, New Caledonia/99 and Brisbane/07. Pre-vaccination sera showed greatest reactivity to Texas/91 in both controls and LRs with GMT of 137 and 40, respectively. Pre-vaccination titres to Texas/91 were higher than those observed to 2009 H1N1pdm.

After pandemic H1N1 vaccination, (D21) sera cross-reacted with all the seasonal H1N1 strains in controls with seroprotective titres in 80–100% of subjects. Back-boosting, with HI titres greater or comparable to those to H1N1pdm, was induced for Texas/91 and Taiwan/86 with GMTs of 1,841 and 618, respectively. In comparison to controls, cross-reactive antibodies in LRs were significantly lower to 4 of the 6 strains; USSR/77, Brazil/78, Taiwan/86 and Texas/91 (*P*<0.05). At D21, the GMTs to USSR/77 and Brazil/78 were low at 23 and 33, respectively, whereas for the 4 other seasonal H1N1 strains titres were >40 (range: 69–442). Seroprotection was lower in LRs (42–92%) compared to controls, with the highest seroprotective titres observed for the Texas/91 strain. In summary, these results show that H1N1 cross-reactivity of post-H1N1pdm vaccination sera to seasonal H1N1 strains is reduced in LRs.

### HA stalk-specific IgG antibodies dominate the response to pandemic H1N1

We dissected the specificity of the antibodies to the different HA domains, which may have different functions in controlling infection. Sera were evaluated for antibodies specific for the H1N1pdm whole HA, HA1 (head domain) and chimeric HA construct, cH6/1 (stalk domain).

**Figure 3:**
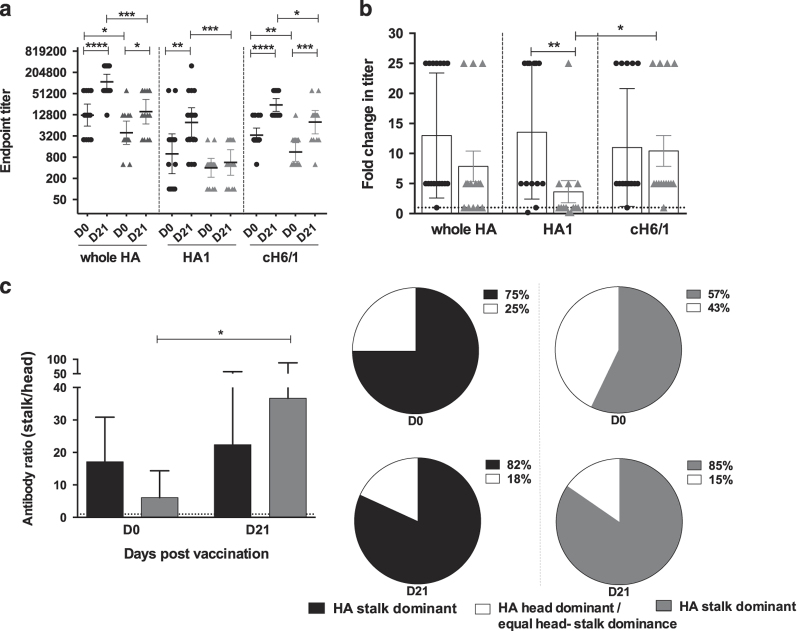
Hemagglutinin-specific IgG antibody responses. (**a**) IgG titres specific to the whole HA (Cal09), HA head domain (HA1) and stalk domain (CH6/1) at D0 and D21 after vaccination in controls (black circles) and low responders (grey triangles). (**b**) Fold change in specific IgG titres calculated as D21 titres÷D0 titres. (**c**) Bar chart shows the ratio of HA stalk-specific antibodies to HA head-specific antibodies at D0 and D21. Bars show mean and error bars show s.d. Pie charts with the proportion of controls (black) and LRs (grey) who have stalk dominant IgG antibody responses. The white pieces of the pie show the proportion of HCW with HA head dominated or equal HA head and stalk dominated IgG response. *P<0.05; **P<0.01; ***P<0.001, ****P<0.0001.

Pre-vaccination, antibodies specific to the whole HA were significantly lower in LRs compared to controls (*P*<0.05). Vaccination resulted in an increase in these antibodies, however to a lesser magnitude than in controls (Figure 3a). The antibody levels induced by vaccination were significantly lower in LRs compared to controls to both the HA head and the stalk domains. In controls, both the HA head and stalk-specific antibodies increased significantly after vaccination (*P*<0.01). However, in the LRs only stalk-specific antibodies increased significantly (*P*<0.001), whereas HA head-specific antibodies remained low. The fold increase in HA stalk-specific antibodies was comparable between the two groups, whereas controls had a higher fold increase in HA head-specific antibodies (*P*<0.01) (Figure 3b). HA stalk-specific antibodies dominated the response pre- and post-vaccination in both groups (Figure 3c). There was a significant increase in the proportion of HA stalk antibodies at D21 in comparison to pre-vaccination ratios in LRs (*P*<0.05). Pre-vaccination, 75% of controls had HA stalk dominant IgG antibodies, whereas at D21, 82% had a HA stalk dominant IgG antibodies. However, in LRs, there was an increase from 57 to 85% in number of participants with stalk dominant antibody responses at D0 and D21, respectively (Figure 3c).

### Revaccination of LRs boosts the HA head-specific antibodies

**Figure 4:**
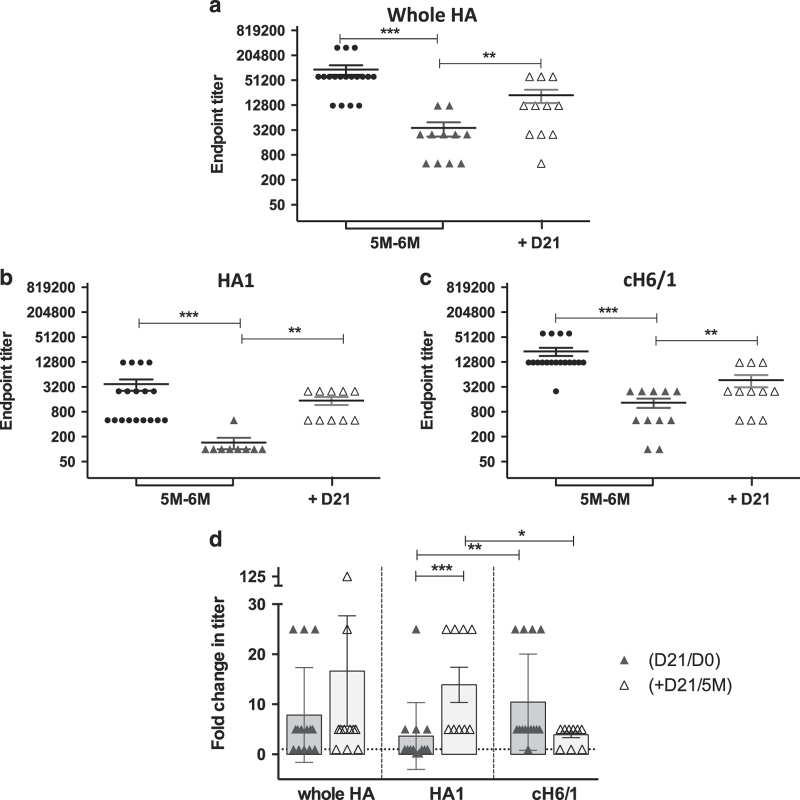
Hemagglutinin-specific IgG antibody responses following revaccination. IgG titres specific to the (**a**) whole HA, (**b**) HA head domain and (**c**) HA stalk domain at 6M in controls (black circles) and 5M (revaccination) (grey triangles) and 21 days after revaccination (+D21) (open triangles) in LRs. (**d**) The bar chart shows fold induction of IgG titres at D21 post-vaccination over D0 titres. Mean fold induction is shown as bars and grey triangles represent each individual’s fold induction after the first vaccination (D21/D0), whereas fold induction 21 days after revaccination is shown in open triangles (+D21/5M). **P<0.01; ***P<0.001.

Next, we analysed the kinetics of the HA-specific IgG antibodies in LRs following revaccination with the AS03 adjuvanted monovalent pandemic H1N1 vaccine. After revaccination, the HA head was no longer novel so we expected a boost of the response to HA head domain. As we did not collect sera from controls at the time of LR revaccination (5M), we used sera collected at 6M for comparison purposes. IgG antibodies specific to the whole HA where maintained for at least 6M in controls but not in LRs where they decreased to pre-vaccination levels. However, revaccination of LRs resulted in a boost in the whole HA-specific antibodies (*P*<0.01) (Figure 4a).

In controls, the HA head-specific IgG antibodies decreased to pre-vaccination levels by 6M. Similarly, in LRs, HA head-specific IgG antibodies returned to pre-vaccination levels at the time of revaccination (5M). The level of these HA1 specific IgG antibodies was significantly lower in the LR group at 5M (*P*<0.001) (Figure 4b). In contrast to HA head-specific antibodies that decreased by 6M post-vaccination, HA stalk-specific antibodies were maintained for at least 6M in controls. However, in LRs, HA stalk-specific antibodies were significantly lower than controls (*P*<0.001) and decreased to pre-vaccination levels 5M post-vaccination (Figure 4c).

Revaccination boosted both the HA head and HA stalk-specific IgG response by D21 (*P*<0.01). After the first vaccination, the IgG titres increased mostly to the HA stalk, in contrast revaccination induced antibody responses mostly to the HA head (Figure 4d). Twenty-one days after the first vaccination, there was higher fold increase in HA stalk-specific IgG than HA head-specific IgG titres. However, following revaccination, there was a significantly higher fold increase in IgG titres to the HA head than after the first vaccination (*P*<0.001).

### HA stalk-specific IgG antibodies show avidity superior to HA head-specific antibodies

**Figure 5:**
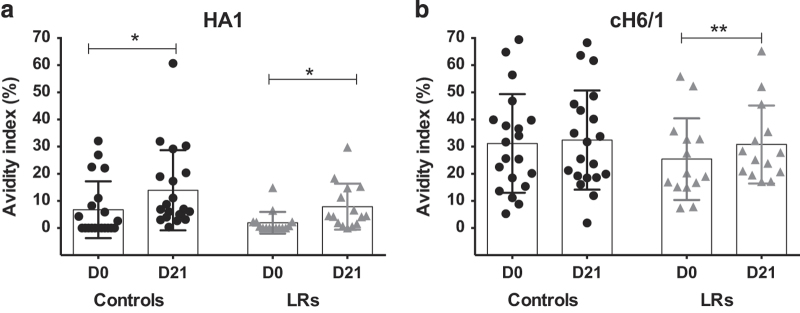
Hemagglutinin-specific IgG antibody avidity. Avidity of (**a**) HA head domain specific and (**b**) HA stalk-specific IgG at D0 and D21 post-vaccination. Avidity index was calculated as the percentage of HA-specific IgG antibodies remaining bound after 1.5 nmol/l NaSCN treatment measured as (absorbance of treated samples÷absorbance of untreated samples)×100%. *P<0.05; **P<0.01.

To evaluate the quality of the antibodies in the controls and LRs, we measured the avidity of HA head and HA stalk-specific IgG antibodies in these two cohorts of HCW. The avidity of HA-specific IgG antibodies was measured in an avidity enzyme-linked immunosorbent assay (ELISA) using NaSCN as a chaotropic agent. Untreated sera were compared to those treated with 1.5 mol/l NaSCN, and the percentage of bound IgG antibodies remaining after 1.5 mol/l NaSCN treatment was calculated. At baseline, the avidity of head-specific antibodies was low. The avidity of head-specific antibodies increased significantly in both controls and LRs following vaccination (*P*<0.05) (Figure 5a). In controls, the avidity index increased from a mean of 3.02–7.11% at D21. In LRs, an increase from a mean of 1.95–8.14% was observed post-vaccination.

The avidity of HA stalk-specific antibodies was significantly higher than that of HA head-specific antibodies at both time-points (Figure 5b). In controls, the % of IgG antibodies remaining bound after NaSCN treatment was 25.74% (range: 5.3–69%) at baseline. Vaccination resulted in a significant increase in the avidity of HA stalk-specific IgG antibodies in LRs only. The avidity index of the IgG antibodies increased from 25.40% (range: 7.46–55.92%) at baseline to 30.82% (range: 17.13–65.26%) at D21 post-vaccination in LRs (*P*<0.01). Despite, the quantity of the stalk-specific antibodies being lower in LRs compared to controls, the antibodies displayed equivalent avidity at both time-points.

### HA stalk-specific antibodies can neutralise virus in the absence of HI antibodies

**Figure 6:**
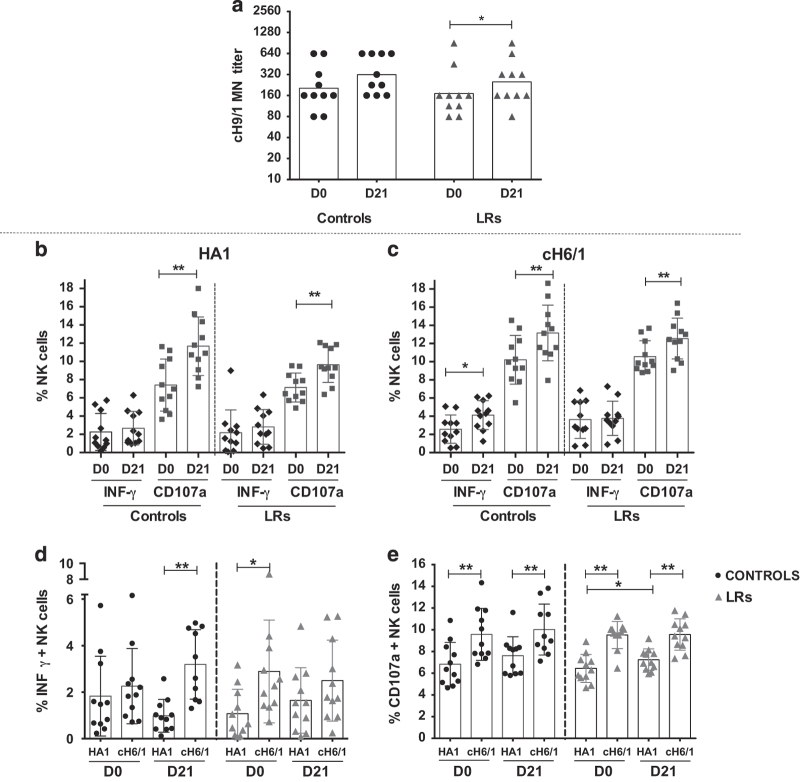
Hemagglutinin-specific antibodies functionality in neutralization and ADCC. (**a**) H1 HA stalk-specific neutralizing antibodies. Geometric mean titres of neutralizing antibodies against cH9/1 N3 virus with H1 HA stalk domain, irrelevant neuraminidase and H9 HA head domain in 10 controls (circles) and 10 low responders (triangles). The frequency of INF-γ expression and CD107a by NK cells to (**b**) HA head domain (HA1) and (**c**) stalk domain (CH6/1) in the presence of sera diluted 1:10 at D0 and D21 after vaccination in 10 controls and 10 low responders. (**d**) INF-γ and (**e**) CD107a expression mediated by HA head-specific antibodies and HA stalk-specific antibodies was compared for each sample sera standardized in an ELISA to give OD of 0.7±0.2. *P<0.05; **P<0.01.

To assess the *in vitro* functionality of H1 HA stalk-specific antibodies, we performed a microneutralization assay with a virus that expresses a cH9/1 HA and an irrelevant N3 neuraminidase. The HA stalk domain of this virus is derived from H1, and the HA head domain is from H9. Subjects in this study are expected to be naive to the avian H9 and N3 in this virus. No standard virus neutralization titre for 50% protection has been recognised. However, a previous study in H1N1pdm-infected adults showed that the MN titre was generally twofold higher than the HI titre when the HI titre was ⩽160.^16^ We therefore used a threshold of 80 to define protective titres. H1 HA stalk-specific neutralising antibodies were detected pre-vaccination when the majority of controls (50%) and LRs (93%) had HI titres<40 (Figure 6a). These H1 HA stalk-specific neutralising antibodies were present in all subjects with GMT of 204 and 171.5 in controls and LRs, respectively. Vaccination resulted in a significant increase in HA stalk-specific neutralising antibodies in LR (*P*<0.05) but not in controls.

### HA stalk-specific antibodies are better mediators of natural killer (NK) cell activation

As the HA-specific antibody response was dominated by HA stalk-specific antibodies, we wanted to determine the functionality and possible protective mechanisms of these antibodies, especially in LRs where HA head-specific antibodies were low. We therefore assessed the ADCC induced by HA head and HA stalk-specific antibodies using an NK activation assay measuring CD107a (degranulation) and INF-γ expression by flow cytometry. The gating strategy for flow cytometry is shown in Supplementary Figure S1. We used sera diluted 1:10 rather than endpoint titres as a positive correlation between NK cell activation at a single sera dilution of 1:10 and NK activation end point titre has been previously shown.^17^ Pre-vaccination, NK cell activation was mediated by both HA head and HA stalk-specific antibodies and the levels of NK cell activation were comparable between LRs and controls. Vaccination resulted in increased NK cell activation with a significant increase in head and stalk antibody-mediated CD107a expression being observed for both LRs and controls (*P*<0.01)(Figure 6b,c). However, the levels of INF-γ expression mediated by HA head-specific antibodies did not increase for either LRs or controls. In contrast to controls who had an increase in INF-γ expression at D21, no change in HA stalk antibody-mediated INF-γ expression was detected in LRs (Figure 6c).

In order to make a direct comparison of the levels of head versus stalk antibody-mediated NK cell activation, we standardized sera in an ELISA assay to give an optical density (OD) of 0.7±0.2. In controls, pre-vaccination HA head and HA stalk-specific antibodies induced similar NK cell expression of INF-γ (Figure 6d). However, post-vaccination, HA stalk-specific antibodies induced significantly higher INF-γ expression than head-specific antibodies in controls (*P*<0.01). In contrast, LRs’ HA stalk-specific antibodies induced significantly higher INF-γ expression at D0 than head-specific antibodies (*P*<0.05), whereas at D21 the INF-γ expression levels were maintained. Pre-vaccination, HA stalk-specific antibodies induced significantly higher NK cell CD107a expression than HA head-specific antibodies in both LRs and controls (Figure 6e). Post-vaccination, only HA head-specific antibodies from LRs had an increased ADCC activity as measured by CD107a expression. Despite no increase in CD107a expression at D21, the HA stalk-specific antibodies maintained a significantly higher ADCC induction than HA head-specific antibodies in both LRs and controls (*P*<0.01). Furthermore, there was no significant difference in the ADCC mediated by both head and stalk-specific antibodies between LRs and controls. Interestingly, there was no correlation between NK cell activation and HI titres in controls (data not shown). However, in LRs, HI titres negatively correlated with NK cell INF-γ expression mediated by both HA head and HA stalk antibodies (*P*<0.01). This suggests that H1N1pdm vaccination induced HA-specific antibodies that can mediate FcγR-dependent NK cell activation, regardless of HI antibodies.

## Discussion

The H1N1pdm virus contained a novel HA head domain that was different from the pre-pandemic seasonal H1 viruses. Pandemic vaccination induced antibodies that were directed towards the immunosubdominant conserved epitopes on the HA stalk domain.^13,14^

In the current study, we aimed to investigate and to increase understanding of the HA-specific IgG responses of LR HCW following 2009 pandemic influenza A (H1N1) vaccination. We used chimeric HA constructs to differentiate between the response to the HA head and stalk domains as these antibodies may have different functions in controlling influenza infection. Influenza specific responses are commonly measured by the HI assay. HI antibodies are a correlate of protection, and are mostly directed to the HA head domain and do not necessarily reflect the entire spectrum of vaccine induced antibodies. We demonstrated that LRs IgG antibodies were quantitatively inferior to controls but qualitatively similar.

We showed that a single dose of AS03 adjuvanted pandemic H1N1 vaccine elicited a significant HI response in controls that was maintained by 3M post-vaccination, whereas in LRs the response was lower and waned by 3M. The lower HI response may be attributed to the lower quantities of HA head-specific antibodies in LRs as antibodies measured by the HI assay are predominantly HA head-specific.

Back-boosting, where a substantial response to older viruses is induced, depending on pre-exposure has been recently described for both H3N2 and H1N1 viruses.^18,19^ Li *et al.*^18^ showed sera from H1N1pdm-infected people had considerable cross-reactivity with H1N1 strains from 1984 to 1994. They showed that the back-boost for H1N1 was due to a shared epitope in the head domain at H1N1pdm and most seasonal H1N1 strains from 1983 to 1996. HAs of most seasonal H1N1 between 1983 and 1996 contained a K133 amino acid at Sa antigenic site of HA, but not H1N1 viruses before 1983 or after 1996. Our results are in agreement with this as we showed back-boost of HI against viruses up to Texas/91 but not much against NC/99 and Brisbane/07. LRs had a lower back-boost of cross-reactive HI antibodies to pre-pandemic, seasonal H1N1 strains. This may be due to differences in exposure history to different H1N1 strains or due to limited induction of HI antibodies that bind to shared epitopes on the HA of pandemic and seasonal H1N1 strains. Moreover, the pandemic vaccine may have elicited antibodies with broader specificities that bind the same epitopes as antibodies induced by seasonal H1N1 strains as well as to additional epitopes. Despite the lower HI antibodies detected in the LR cohort, HA stalk-specific antibodies may also provide an alternative method of protection.

We tested for the HA domain binding of IgG antibodies and found that HA stalk-specific antibodies were lower pre-vaccination in LRs, possibly due to differences in the priming or infection history between controls and LRs or due to the characteristics of the HA-specific memory previously generated. Even though pre-vaccination HA stalk-specific antibodies were lower, a single dose of H1N1pdm vaccine elicited significant stalk-specific antibodies more efficiently than HA head-specific antibodies. However, 5M post-vaccination, HA head and stalk-specific antibodies in LRs had decreased and were significantly lower than those in controls. Although none of the HCW reported H1N1pdm infection, it cannot be ruled out that some controls had exposure or subclinical infection between the 5M and 6M interval. Alternatively, the lower antibodies in LR could be explained by poor antibody maintenance in this cohort. The decreased antibody titres may not necessarily mean that the HCW were no longer protected, as factors other than antibody titres may be important in long-term protection.

These findings raise questions as to: (i) why the conserved HA stalk domain shows superior immunogenicity after H1N1pdm vaccination, whereas after seasonal vaccination the HA head-specific response dominates^20,21^; and (ii) the effect of homologous boosting on HA domain binding. We found a significantly higher boost in head than stalk-specific antibodies following revaccination of LRs. This is in line with previous reports following H1N1pdm and H5N1 vaccination.^22,23^ Ellebedy *et al.* demonstrated that the first dose of H5N1 vaccination elicited a stronger stalk-specific response than head-specific response. However, booster vaccination resulted in a vigorous head-specific response and marginal increase in stalk-specific antibodies.^23^ These findings support the theory that at the time of the first vaccination, vaccinees had negligible pre-existing memory B cells specific to the immunodominant HA head domain in comparison with stalk-specific ones. The strong stalk-specific antibody response likely reflects the reactivation of stalk-specific memory B cells generated by previous seasonal H1N1 infections. Accordingly, memory B cells specific for the immunosubdominant HA stalk were recruited and reactivated in the absence of competition from memory B cells specific for the immunodominant HA head domain. A primary antibody response was also induced to the HA head domain resulting in the increase in plasmablasts and memory B cells generation. Upon booster vaccination, the recently generated head-specific memory B cells out-competed the stalk-specific memory B cells.^24^

The quality of antibodies is important for their functionality. In our analysis, HA stalk-specific antibodies not only dominated the response but also displayed superior avidity to head-specific antibodies. These results are in agreement with the findings of He *et al.*,^20^ who showed that plasmablast-derived polyclonal antibodies from elderly (>70 years) after H1N1pdm vaccination had higher avidity than those from young (18–32 years) and that HA2 specific antibodies had higher avidity than HA1 specific antibodies. The higher avidity of stalk-specific antibodies could be explained by antibody secreting cells producing these antibodies being derivatives of memory B cells from previous H1N1 encounters that have gone through several rounds of selection and affinity maturation.^25,26^As the 2009 pandemic H1N1 contained a novel head, different from the pre-pandemic seasonal H1N1 viruses, most of the HA head-specific antibodies would have been generated from antibody secreting cells from a primary immune response that contain few somatic mutations, explaining the lower avidity of HA head-specific antibodies. It has been shown that broadly cross-reactive HA-specific antibodies that exhibit high levels of somatic hypermutation are induced after H1N1pdm vaccination or infection.^13,27^ Analysis of the somatic mutation status of the immunoglobulin genes from the memory B cells or plasmablasts specific for the HA head and stalk domains is required to give insight into the origin of the response in our cohort of HCW. These high-avidity HA stalk-specific antibodies may have an important role in protecting these LRs in the absence of HI antibodies. However, a correlate of protection for these antibodies remains to be determined.

We showed that high titres neutralising HA stalk-specific antibodies were present in all subjects pre-vaccination, when HI antibodies were absent or low in the majority of the HCW. The neutralising stalk-specific antibodies were maintained in controls and only increased significantly in LRs after vaccination. These neutralising stalk-specific antibodies could be protective regardless of the HI titre and could have a significant role in protecting the low responders who have significant quantities of HA stalk-specific antibodies.

In addition to virus neutralization, HA-specific antibodies can bind to infected cells and activate NK cells through FcγR resulting in lysis of target cells and secretion of cytokines like INF-γ.^17^ In this study, we showed that ADCC antibodies were present pre-vaccination when the majority of the subjects were HI seronegative and these ADCC antibodies were mostly directed towards the HA stalk domain. These pre-existing ADCC antibodies may assist in clearance of H1N1pdm virus infection. Post-vaccination, the HA stalk-specific antibodies were better mediators of ADCC than HA head-specific antibodies. This difference in the ADCC activity between HA head and stalk-specific antibodies may be attributed to the relative difference in affinities of these antibodies for HA. Despite LRs having lower post-vaccination HI titres and lower HA-specific IgG titres than controls; their antibodies had comparable ability to mediate NK cell activation. We found no correlation between HI titre and NK cell activation in controls, whereas in LRs INF-γ expression negatively correlated with HI titres. Our results are similar to a previous report that found no correlation between HI titres and HA-specific ADCC antibodies.^17^ However, these authors characterised the response induced by antibodies that bound to the whole HA protein, whereas we dissected between HA head and stalk-specific antibodies. Of note, the HI and ADCC assays measure different aspects of antibody function. HI assay assesses the binding of the antibody through its Fab region to antigen, whereas the ADCC assay assesses the NK activation by antibodies bound through their Fc region. Fc–FcγR interactions are required for the protection by HA stalk-specific antibodies suggesting a role of ADCC antibodies in protection.^28–30^ Furthermore, cross-reactive ADCC antibodies to influenza virus have been reported in the absence of neutralising antibodies.^31^ Thus, it is likely that HA stalk-specific antibodies may provide alternative protection in this cohort of LRs where HA head-specific antibodies are low and induced short-term protection as measured by the HI assay. This underlines a possible limitation of using HI titre as a sole predictive correlate of protection.

In summary, the characterisation of the HA stalk-specific antibodies denotes an important step in understanding the protective capacity of these antibodies. Further analysis of the HA stalk-specific antibody repertoire and function would facilitate rational vaccine design. In LRs where the HI responses are poor, vaccination strategies could potentially aim to induce HA stalk-specific antibodies. The lower quantity of HA-specific antibodies could be compensated for by the higher avidity of HA stalk-specific antibodies that have better ADCC functionality as well as neutralising capacity. Enhancing the amount of HA stalk-specific antibodies elicited by vaccination or booster vaccination should be considered in LR HCW. These antibodies could provide broad cross-protection and could be used for immunological priming of the general population to quickly respond to a future pandemic influenza threat.

## Materials and Methods

### Study design and participants

The HCWs were vaccinated in October 2009 at the Haukeland University Hospital (HUH, Bergen, Norway) with a single dose of the monovalent pandemic H1N1 vaccine (Pandemrix) adjuvanted AS03 (GlaxoSmithKline (GSK), Wavre, Belgium).

Thirty-six HCW were retrospectively selected by their HI antibody response at 3M after pandemic H1N1 vaccination. On this basis, a cohort of 14 LRs who failed to respond or maintain a protective HI antibody response (titre⩾40) at 3M after vaccination were selected. As a control group, 22 HCW who maintained a protective HI antibody response were selected. LRs were offered a second dose of vaccine, and 12 participants were revaccinated 5M later. Sera were collected at vaccination, day 21 (D21), 3M, and 6M post-vaccination in controls. In revaccinated LRs, additional sera were collected at revaccination (5M) and 21 days later (Figure 1a). The inclusion and exclusion criteria for this study are described elsewhere.^7^ All participants provided written informed consent before inclusion in the study, which had ethical and regulatory approval (ClinicalTrials.gov NOT01003288).

### HI assay

Serum samples were treated with receptor destroying enzyme and run in the HI assay twice in duplicate using turkey red blood cells as previously described.^7^ HI responses were analyzed against the homologous pandemic H1N1 virus strain, A/California/07/09, and against six prototype seasonal H1N1 strains; A/USSR/90/77, A/Brazil/11/78, A/Taiwan/1/86, A/Texas/36/91, A/New Caledonia/20/99 and A/Brisbane/59/07. Seroprotection was defined as an HI titre ⩾40. Titres<10 were assigned a value of 5 for calculation purposes.

### Anti-HA IgG ELISA

Sera were evaluated in duplicate for IgG antibodies.^32^ The plates were coated with influenza whole HA, HA1 (A/California/06/2009(H1N1)) hexahistidine-tagged (eEnzyme, IA-01SW-005P) or cH6/1, a chimeric HA (cHA) that combines H1 stalk domain with globular head domain derived from H6 influenza A virus.^33^ The antibody concentrations were calculated as endpoint titres that were determined when the reactivity of the diluted sample reached background levels.

### Anti-HA IgG avidity ELISA

Sera were evaluated for avidity of antibodies against influenza HA1 (A/California/06/2009(H1N1)) hexahistidine-tagged (eEnzyme, Gaithersburg, MD, USA) and cH6/1.^34^ Sera were first diluted in duplicate to the appropriate OD of 0.7±0.3 in a direct ELISA and 1.5 mol/l sodium thiocyanate (NaSCN) (Sigma, St Louis, MO, USA) was added 1 h after the sera, followed by 1 h incubation. The avidity index calculated as: (OD_450_ treated serum/OD_450_ untreated serum)×100%.

### Virus neutralization assay

#### Expansion of MDCK cells

Madin-Darby Canine Kidney (MDCK) (ATCC, Manassas, VA, USA, CCL-34) were cultivated in DMEM supplemented with 10% fetal bovine serum (FBS) and 1× Penicillin/Streptomycin. MDCK cells in log-phase growth were plated in 96-well plates such that they are 70–90% confluent at the time of inoculation. Serum samples were heat-inactivated at 56 °C for 30 min and run in duplicate. The serum samples were then diluted 2 fold in virus growth medium containing Dulbecco’s Modified Eagle’s Medium with tosyl phenylalanyl chloromethyl ketone-trypsin, 0.14% bovine serum albumin, 100 units/ml penicillin, 100 μg/ml streptomycin and 0.25 μg/ml amphotericin B. Chimeric 9/1N3 virus was diluted to 50% tissue culture infectious dose (TCID50) of 100 per 50 μl in virus growth medium. Fifty microliters of diluted sera was incubated with 50 μl of virus for 1 h at 37 °C. MDCK cells were washed once with phosphate-buffered solution (PBS) and 100 μl of serum-virus mixture was added to the cells. Cells were incubated at 37 °C for 1 h then washed once with PBS before 50 μl of diluted serum and 50 μl of virus growth medium were added to each well. Infected MDCK cells were incubated for 72 h at 37 °C. Fifty microliters of the supernatant was transferred to a 96-well V bottom plate and 50 μl of 0.7% turkey red blood cells added. Hemagglutination activity was measured to detect the endpoint of agglutination.

### ADCC NK cell activation assay

The ADCC assay measuring intracellular NK cell IFNγ and CD107a expression was conducted as previously described with minor modifications.^35^ Briefly, 96-well plates were coated overnight at 4 °C with 1 μg/ml HA1 (A/California/06/2009(H1N1)) 6×His tagged (eEnzyme, USA) or chimeric cH6/1 in PBS. Plates were washed with PBS and incubated with heat-inactivated human sera for 2 h at 37 °C. After washing, 10^5^ CD16 176v NK-92 cells (mycoplasma-free, human NK cell line expressing high affinity 176V variant CD16 receptor) (kindly provided by Fox Chase Cancer Center, Philadelphia, PA, USA) were added per test well. As a negative control for each sample, NK-92 cells (lacking expression of CD16) were added to an additional well. The cells were incubated for 16 h at 37 °C with anti-CD107a-AF488 antibody (Biolegend, San Diego, CA, USA, 328610), Brefeldin A (5 μg/ml, BD) and monensin (5 μg/ml, BD). Cells were stained with LIVE/DEAD Fixable Aqua dead cell staining kit (Invitrogen, Carlsbad, CA, USA), anti-CD3-PE CF594 (BD, Franklin Lakes, NJ, USA, 562280) and anti-CD56-APC (BD, 555518) before intracellular staining with anti-IFN-γ-BV-421 (Biolegend, 502532). Cells were acquired on BD Fortessa (San Jose, CA, USA). Data analysis was done using FlowJo version 10 (treeStar, Ashland, OR, USA).

### Statistics

Two-tailed unpaired nonparametric Mann–Whitney tests and paired nonparametric Wilcoxon tests were performed using GraphPad Prism 6 (Graphpad, La Jolla, CA, USA). A *P*-value<0.05 was considered statistically significant.
