# Supplementary figures and images for: Dissecting the hemagglutinin head and stalk-specific IgG antibody response in healthcare workers following pandemic H1N1 vaccination
Source: NPJ Vaccines. 2016 Jul 28;1:16001–. doi: 10.1038/npjvaccines.2016.1 (PMC5707877; doi:10.1038/npjvaccines.2016.1)

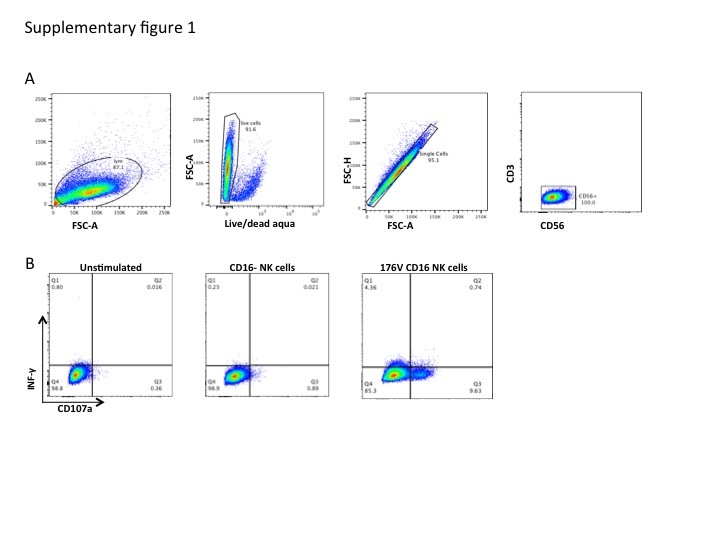

Supplement: Supplementary Figure S1 [file npjvaccines20161-s1.jpg]
